# Usefulness of computed tomography for hospitalized adult patients with fever to investigate cause of fever: single-center, retrospective cohort study

**DOI:** 10.1007/s11604-021-01117-5

**Published:** 2021-05-01

**Authors:** Fumiko Hamabe, Takero Terayama, Ayako Mikoshi, Wakana Murakami, Kohei Yamada, Shigeyoshi Soga, Hiroshi Shinmoto

**Affiliations:** 1 Department of Radiology, National Defense Medical College, Namiki 3-2, Tokorozawa, Saitama Japan; 2 Department of Traumatology and Critical Care Medicine, National Defense Medical College Hospital, Namiki 3-2, Tokorozawa, Saitama Japan; 3 Department of Diagnostic Radiology, Keio University School of Medicine, Shinanomachi 35, Shinjuku, Tokyo, Japan; 4 Department of Radiology, Division of Radiology, Showa University Graduate School of Medicine, 1-5-8 Hatanodai, Shinagawa-ku, Tokyo, Japan

**Keywords:** Computed tomography, Fever, Inpatients, Diagnostic imaging, Appropriate use

## Abstract

**Purpose:**

To verify that physicians’ presumptive diagnosis is the most significant factor for finding any signs related to the cause of fever on computed tomography (CT) images.

**Materials and methods:**

This single-center retrospective cohort study included patients (age ≥ 16 years) who underwent CT to investigate the cause of fever between January 1, 2014, and August 31, 2016. Patients who underwent surgical procedures were excluded. The primary outcome was the presence of suspicious CT findings related to the cause of fever. We performed univariate and multivariate logistic regression analyses, adjusted for CT contrast agent use, quick sequential organ failure assessment score > 1, and C-reactive protein level.

**Results:**

We enrolled 171 patients, of which 57 had CT findings, and 114 did not. Multivariate logistic regression analyses demonstrated a significant difference for the presence of a presumptive diagnosis by the attending physician (odds ratio, 4.99; 95% confidence interval 2.31–10.76; *p* < 0.01), but not for other covariates, including C-reactive protein.

**Conclusions:**

In hospitalized patients with fever, an attending physicians’ presumptive diagnosis is associated with the presence of fever-related CT findings. Improving the quality of the diagnostic assessment before the CT scan may lead to more appropriate CT imaging use.

**Supplementary Information:**

The online version contains supplementary material available at 10.1007/s11604-021-01117-5.

## Introduction

Computed tomography (CT) is a versatile diagnostic imaging tool used in various clinical settings. As Japan has more CT scanners relative to the population size than any other country (111 CT units per 1 million people) [1], clinicians tend to frequently use CT scans to investigate the cause of a fever. Moreover, with the worldwide outbreak of coronavirus disease (COVID)-19 in 2020 and reports of ground-glass opacities on chest CT images even in asymptomatic patients [2, 3], more hospitals have tended to perform CT imaging. On the other hand, the increased use of CT scans may cause harm, such as increased cancer risk from radiation or increased medical costs from overdiagnosis and overtreatment. Indeed, the Nuclear Safety Research Association reported in 2008 that Japanese people were exposed to more radiation, especially from that in medical applications such as CT [4]. Therefore, appropriate use of CT will be more required in the future.

To evaluate the usefulness of CT scans in the investigation of causes of fever, several categories of factors must be considered. First, the urgency determined by a patient’s condition affects the usefulness. Patients with severe hypoxia or hemodynamically unstable status require immediate interventions; therefore, CT would be recommended as part of the initial treatment, because a delay in intervention can affect their prognosis. Second, patient factors such as a present illness or past medical history may affect the usefulness of this imaging modality. For example, aged patients with dementia often have less prominent chief complaints and physical findings, even if they have an infection. In these situations, clinicians perform CT scans more frequently to decide about the treatment. Third, the physician’s skills to assess a patient with fever has an influence. Higher skilled physicians can provide a clearer indication for the CT scan; therefore, a suspicious finding as the cause of fever would be detected more easily on CT images. Fourth, circumstance or cultural background also affects the usefulness. In Japan, the large number of CT units provides such good accessibility to CT scans that physicians tend to use this imaging technique more frequently. Moreover, physicians sometimes defensively perform CT scans to avoid potential litigations. Thus, the usefulness of CT scans for the investigation of the cause of a fever may vary depending on clinical settings.

Patients are categorized mainly into two groups: outpatients, including emergency settings, and inpatients. In the case of outpatients, physicians usually have less clinical information and more time constraints on examining the patients than in the case of inpatients. Especially in emergency situations such as unstable vital signs, the value of CT scans can relatively increase. Conversely, when treating inpatients, physicians should more carefully evaluate the necessity of a CT scan, because they have more information about baseline characteristics and clinical course at hand and have more diagnostic tools available compared to the treatment of outpatients.

Although several studies have reported on the usefulness of CT scans for various infectious diseases, no study has evaluated the usefulness of CT scans in the investigation of the cause of fever [5, 6]. Therefore, as a first step to evaluate the usefulness of CT scans for investigating the cause of a fever, we performed a retrospective cohort study focusing on inpatients. Specifically, we sought to verify the hypothesis that the suspected diagnosis by the treating physician is more significant than blood sampling data to identify on CT a suspicious finding as to the cause of a fever.

## Materials and methods

### Study design

This single-center, retrospective cohort study was conducted in the National Defense Medical College (NDMC) Hospital, Saitama, Japan, a 638-bed tertiary teaching hospital with approximately 17,000 annual admissions, in accordance with the ethical guidelines of the Declaration of Helsinki. This study was approved by the Ethics Committee of NDMC, license number 4258.

### Patients

Patients eligible for this study were inpatients with suspected infection, treated in the NDMC hospital between January 1, 2014, and August 31, 2016, aged 16 years or older, who received a CT scan to investigate the source of infection. Exclusion criteria were as follows: pregnancy, CT was performed at admission, CT was performed as a follow-up for a known infectious disease, and CT was performed within 30 days after surgical intervention. We judged the known infectious disease considering the medical records or comments of CT-ordering sheet.

### Outcome

The primary outcome was whether suspicious findings regarding the cause of the fever were identified on CT images (presence of suspicious findings). Two radiologists (F.H and A.M) read all CT images of the enrolled patients and evaluated them regarding the presence of suspicious findings. The radiologists judged findings as “suspicious” according to the criteria shown in Table 1. They also used other clinical information provided by the medical records of patients for this assessment.

**Table 1:** CT findings that may constitute a “suspicious finding”

Cause of fever	CT finding
Infectious disease	
Lung	Centrilobular nodules/branching structures Segmental distribution Airspace consolidation Ground-glass opacity Pulmonary nodules Cavitation
Mediastinum	Abscess Fat stranding Pleural thickening Pleural effusion
Abdomen/pelvis	Abscess Enlargement of an organ Disproportionate fat stranding Abnormal intraabdominal fluid
Carcinoma-related fever	Rapid increase or enlargement of the tumor Necrosis of the tumor Rupture of the tumor
Other	Neither infectious disease nor carcinoma-related fever (i.e., vasculitis, autoimmune disorders)

*CT* computed tomography

### Variables and measurements

We examined covariates that may affect the outcome based on the clinical perspective. Clinical data were collected from medical records of the patients and included age, sex, past medical history, any immunosuppressive disease, vital signs, quick sequential organ failure assessment (qSOFA) score, blood tests [creatinine, C-reactive protein (CRP), white blood cell (WBC) count], use of contrast agents in CT, and intensive care unit (ICU) admission. If these clinical data were not available for the day of the CT scan, the nearest data within 2 days were retrieved. We also assessed based on the medical records whether the attending physician suspected any specific disease before the CT scan, but it was not relevant whether their assessment was consistent with the final diagnosis.

### qSOFA

qSOFA is a new criterion for the rapid identification of patients with suspected sepsis in non-ICU settings. This assessment considers three clinical variables and ranges from 0 to 3 points with 1 point each for systolic hypotension (≤ 100 mm Hg), tachypnea (> 21/min), and altered mentation. A score of 2 or more points is defined as positive, and in this case, the SOFA score is determined to diagnose sepsis. Based on a prior cohort study among non-ICU patients, the predictive validity of the qSOFA score for in-hospital mortality was higher than those of both SOFA and systemic inflammatory response syndrome [7].

### Immunocompromised conditions

Immunocompromised conditions were defined as follows: patients who received any anticancer drugs, oral or intravenous steroid therapy, or any immunosuppressive drugs; patients who had immunosuppressive diseases such as infection with the human immunodeficiency virus HIV or hematologic malignancies.

### Study procedure

The emergency physician (T.T.) extracted a list of eligible patients from the database of the Department of Radiology and obtained all variables from the medical records. Two radiologists (F.H. and A.M.) independently reviewed all CT images and evaluated them according to the following three categories: abnormal (cause of fever can be found on the CT scan), equivocal, and normal. In cases categorized as abnormal, they also classified the type of the cause of fever: infections, neoplasms, and others. Disagreements between the two radiologists were resolved via consensus.

### Data analysis

Continuous variables were summarized as the median and interquartile range (IQR), and categorical variables as frequencies and proportions. Continuous variables with normal distribution, which was assessed by the Shapiro–Wilk test, were compared using the chi-square test. Categorical and continuous variables without normal distribution were compared using the Mann–Whitney *U* test. The effects of covariates (use of contrast agents in CT, qSOFA score > 1, CRP, presence of suspicious findings, the attending physicians’ suspected diagnosis regarding the cause of the fever before the CT scan) on the presence of suspicious findings were assessed in univariate and multiple logistic regression analyses. Subgroup analyses were performed both in patients with immunosuppressive conditions and in patients with infectious causes of fever.

All statistical analyses were conducted with SPSS version 27.0 (IBM Corporation, Armonk, NY, USA). A two-sided significance level of less than 0.05 was used, as were 95% confidence intervals (CIs).

## Results

### Patients

**Fig. 1:**
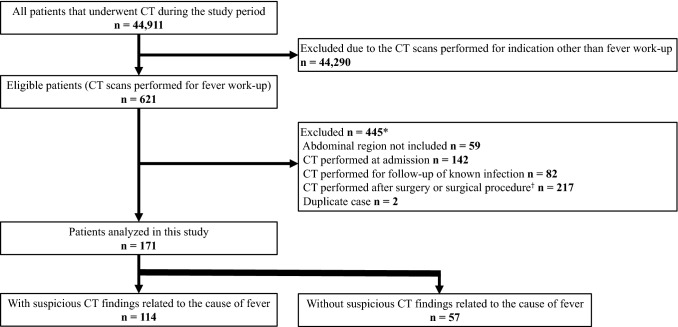
Patient flowchart. *CT* computed tomography. *Differences are due to duplicates. ^†^Evaluated by two radiologists

During the study period, 44,911 patients underwent CT scans. Of these patients, 621 patients underwent CT scans to investigate the cause of a fever. Of these patients, 445 patients were excluded mainly due to the CT scan being performed at admission or in the post-surgery period. Finally, 171 patients were included in the analysis, of whom 114 had some suspicious findings regarding the cause of their fever, whereas 57 did not (Fig. 1). There were no disagreements that were not resolved between the two radiologists (F.H and A.M).

The median age was 71 years (IQR 52.0–74.5 years) in the group with suspicious findings on CT and 65 years (IQR 55.75–77.0 years) in the group without a finding. There was a statistically significant difference between the study groups in CRP levels [6.75 mg/dL (IQR 2.15–10.3 mg/dL) vs 6.2 mg/dL (IQR 3.25–12.75 mg/dL), *p* = 0.027] but not in WBC count [7.95 × 10^3^/µL (IQR, 3.9–9.7 × 10^3^/µL) vs 6.7 × 10^3^/µL (IQR 4.45–12.3 × 10^3^/µL), *p* = 0.08]. In the CT-finding group, a higher percentage of patients had a qSOFA score ≥ 2 (27.3% vs 17.9%, *p* = 0.18) without reaching statistical significance. More attending physicians had a suspected diagnosis regarding the cause of the fever before the CT scan in the CT-finding group compared to the no-CT-finding group (57.3% vs 20.0%, *p* < 0.01). Among the study participants in the CT-finding group, infectious disease as the cause of fever was identified on CT in 86.8% (Table 2).

**Table 2:** Comparison of the characteristics of eligible patients between the two study groups

Characteristics	CT-finding	No-CT-finding	*p*^a^
*N*	114	57	
Age (years)	71 (IQR 55.75–77.0)	65 (IQR 52–74.5)	0.09
Sex, male	82 (71.9%)	43 (75.4%)	0.630
CRP (mg/dL)	6.75 (IQR 3.25–12.75)	6.2 (IQR 2.15–10.3)	0.027
WBC count (× 10^3^/µL)	7.95 (IQR 4.45–12.3)	6.7 (IQR 3.9–9.7)	0.08
Creatinine (mg/dL)	0.87 (IQR 0.61–1.39)	0.76 (IQR 0.63–1.17)	0.43
qSOFA score ≥ 2	30 (27.3%)	10 (17.9%)	0.180
Ward, ICU	2 (1.8%)	0 (0%)	0.600
Immunocompromised conditions^b^	60 (52.6%)	32 (56.1%)	0.660^c^
HIV	4 (3.5%)	1 (1.8%)	0.490
Anticancer drug	36 (31.6%)	20 (35.1%)	0.560
Steroid use	20 (17.7%)	14 (24.6%)	0.290
Immunosuppressive diseases^d^	30 (26.3%)	18 (31.6%)	0.610
Diabetes mellitus	22 (19.3%)	13 (22.8%)	0.420
Use of contrast agents in CT	66 (57.9%)	34 (59.6%)	0.830
Presence of an attending physicians’ estimation regarding a cause of the fever before the CT scan^e^	63 (57.3%)	11 (20%)	0.000
Departments			
Hematology	21 (18.4%)	13 (22.8%)	
Lower digestive surgery	15 (13.2%)	4 (7%)	
Infectious disease and respiratory medicine	13 (11.4%)	4 (7%)	
Nephrology	10 (8.8%)	6 (10.5%)	
Gastroenterology	10 (8.8%)	1 (1.8%)	
Urology	9 (7.9%)	2 (3.5%)	
Upper digestive surgery	8 (7%)	1 (1.8%)	
Hepato-biliary–pancreatic surgery	6 (5.3%)	1 (1.8%)	
Cardiology	5 (4.4%)	5 (8.8%)	
Dermatology	2 (1.8%)	4 (7%)	
Rheumatology	2 (1.8%)	3 (5.3%)	
Obstetrics and gynecology	2 (1.8%)	0 (0%)	
Endocrinology	2 (1.8%)	0 (0%)	
Neurology	1 (0.9%)	4 (7%)	
General medicine	1 (0.9%)	4 (7%)	
Neurosurgery	1 (0.9%)	3 (5.3%)	
Cardiovascular surgery	1 (0.9%)	0 (0%)	
Plastic surgery	1 (0.9%)	0 (0%)	
Emergency department	1 (0.9%)	0 (0%)	
Oral and maxillofacial surgery	1 (0.9%)	0 (0%)	
Orthopedic surgery	1 (0.9%)	0 (0%)	
Otolaryngology	0 (0%)	1 (1.8%)	
Psychiatry	0 (0%)	1 (1.8%)	
Cause of fever			
Infectious disease	99 (86.8%)	–	
Carcinoma-related fever	10 (8.8%)	–	
Other	3 (2.7%))	–	
Multiple causes	2 (1.8%)	–	

*CRP* C-reactive protein, *CT* computed tomography, *HIV* human immunodeficiency virus, *ICU* intensive care unit, *IQR* interquartile range, *qSOFA* quick sequential organ failure assessment, *WBC* white blood cell

^a^Mann–Whitney *U* test or *χ*^2^ test

^b^Defined as follows: patients who received any anticancer drug, oral or intravenous steroid therapy, or any immunosuppressive drugs; patients who had immunosuppressive diseases, such as HIV infection or hematologic malignancies

^c^The value was calculated from the *χ*^2^ test of two groups: one with at least one immunosuppressive condition and one without

^d^This mainly consists of hematologic malignancies

^e^We based this on medical records or CT requests

### Factors associated with detecting any CT findings as to the cause of fever

Multiple logistic regression analyses identified factors associated with CT findings related to the cause of fever. The presence of an attending physicians’ estimation regarding the fever cause before the CT scan was associated with CT findings [odds ratio (OR) 4.99; 95% CI 2.31–10.76; *p* = 0.00; Table 3]. On the other hand, there was no association with the use of contrast agents (OR 0.88; 95% CI 0.43–1.79; *p* = 0.72), qSOFA score ≥ 2 (OR 1.57; 95% CI, 0.66 to 3.72; *p* = 0.30), CRP level (OR 1.04; 95% CI 0.47–1.96; *p* = 0.91), or presence of immunocompromised conditions (OR 0.96; 95% CI 0.47–1.96; *p* = 0.91).

**Table 3:** Multivariate logistic regression analysis of factors associated with the presence of signs for a suspected cause of fever on CT images

Factors	Odds ratio	95% CI	*p*
Use of contrast agents in CT	0.88	0.43–1.79	0.72
qSOFA score ≥ 2	1.57	0.66–3.72	0.30
CRP	1.04	0.98–1.1	0.21
Presence of immunocompromised conditions^a^	0.96	0.47–1.96	0.91
Presence of an attending physicians’ estimation regarding a cause of the fever before the CT scan^b^	4.99	2.31–10.76	0.00

*CI* confidence interval, *CRP* C-reactive protein, *CT* computed tomography, *HIV* human immunodeficiency virus, *qSOFA* quick sequential organ failure assessment

^a^Including anticancer drugs, steroid therapy, immunosuppressive drugs, and immunosuppressive diseases, such as HIV infection and hematologic malignancies

^b^We based this on medical records or CT requests

### Subgroup analysis in patients with immunosuppressive conditions

We also performed a subgroup analysis focusing on patients with immunosuppressive conditions (Table 4). In this analysis, 92 patients were included, of whom 60 had a suspicious CT finding regarding the cause of fever, and 32 did not. Table 4 summarizes the details of the patient characteristics. The CRP value was significantly higher in the CT-finding group compared to the no-CT-finding group [5.4 mg/dL (IQR 3.14–11.65 mg/dL) vs 3.1 mg/dL (IQR 0.95–8.63 mg/dL), *p* = 0.01]. Similar to the analysis in the entire study population, a suspected cause of fever by the attending physician before the CT scan was in this subgroup analysis also associated with detecting fever-related CT findings [30 (52.6%) vs 8 (25.8%), *p* = 0.02].

**Table 4:** Comparison of patient characteristics in subgroups with and without CT findings regarding the cause of fever

Characteristics	CT-finding	No-CT-finding	*p*^a^
*N*	60	32	
Age (years)	71 (IQR 51.25–75)	59 (IQR 49.25–69.75)	0.12
Sex, male	39 (65%)	25 (78.1%)	0.190
CRP (mg/dL)	5.4 (IQR 3.14–11.65)	3.1 (IQR 0.95–8.63)	0.01
WBC count (× 10^3^/µL)	7.6 (IQR 2.98–11.85)	5.4 (IQR 2.3–9.18)	0.17
Creatinine (mg/dL)	0.8 (IQR 0.54–1.12)	0.7 (IQR 0.65–1.02)	0.94
qSOFA score ≥ 2	13 (22.4%)	7 (21.9%)	0.950
Ward, ICU	0 (0.0%)	0 (0.0%)	0.650
Diabetes mellitus	7 (11.7%)	7 (21.9%)	0.270
Use of contrast agents in CT	35 (58.3%)	21 (65.6%)	0.490
Presence of an attending physicians’ estimation regarding a cause of the fever before the CT scan^b^	30 (52.6%)	8 (25.8%)	0.020
Departments			
Hematology	21 (35%)	13 (40.6%)	
Lower digestive surgery	11 (18.3%)	1 (3.1%)	
Infectious disease and respiratory medicine	5 (8.3%)	4 (12.5%)	
Nephrology	5 (8.3%)	3 (9.4%)	
Gastroenterology	0 (0.0%)	1 (3.1%)	
Urology	4 (6.7%)	2 (6.3%)	
Upper digestive surgery	2 (3.3%)	1 (3.1%)	
Hepato-biliary–pancreatic surgery	2 (3.3%)	1 (3.1%)	
Cardiology	0 (0.0%)	1 (3.1%)	
Dermatology	2 (3.3%)	1 (3.1%)	
Rheumatology	2 (3.3%)	2 (6.3%)	
Obstetrics and gynecology	2 (3.3%)	0 (0.0%)	
Endocrinology	1 (1.7%)	0 (0.0%)	
Neurology	1 (1.7%)	1 (3.1%)	
General medicine	1 (1.7%)	0 (0.0%)	
Neurosurgery	0 (0.0%)	1 (3.1%)	
Otolaryngology	1 (1.7%)	0 (0.0%)	
Cause of fever		–	
Infectious disease	50 (83.3%)	–	
Carcinoma-related fever	8 (13.3%)	–	
Other	1 (1.7%)	–	
Multiple causes	1 (1.7%)		

*CRP* C-reactive protein; *CT* computed tomography; *ICU* intensive care unit; *IQR* interquartile range; *qSOFA* sequential organ failure assessment; *WBC* white blood cell

^a^Mann–Whitney *U* test or *χ*^2^ test

^b^We based this on medical records or CT requests

## Discussion

To the best of our knowledge, this is the first study to evaluate the usefulness of CT scans to determine the cause of a fever. Two interesting results were found in this study. First, in the case of inpatients with fever, the attending physician’s presumptive diagnosis before the CT scan was associated with the assessment of CT findings. Second, inflammatory responses, as indicated by CRP levels and qSOFA scores, and immunocompromised conditions had limited effects on the CT scan assessment. Furthermore, infectious diseases accounted for most of the causes of fever.

This study showed that the attending physicians should establish a presumptive diagnosis before using CT scans to further investigate the cause of a fever. Many physicians may have already realized that this result is clinically obvious, but our study confirms it. Several publications have reported on the usefulness of CT in certain infectious diseases, whereas there is no study on the usefulness of CT to investigate the cause of a fever. For example, CT has high sensitivity and specificity in the diagnosis of appendicitis. Terasawa et al. [5] conducted a meta-analysis of the diagnostic accuracy of appendiceal CT in adults and adolescents and showed an overall sensitivity of 0.94 (95% CI 0.91–0.95) and specificity of 0.95 (95% CI 0.93–0.96) in 12 prospective CT studies. Doria et al. [6] conducted a meta-analysis and demonstrated that the pooled sensitivity and specificity values for diagnosis of appendicitis in adults were 0.94 (95% CI 0.92–0.95) and 0.94 (95% CI 0.94–0.96), respectively, in 31 prospective and retrospective trials. On the other hand, CT has lower diagnostic accuracy in acute cholecystitis, and one study reported that the correct diagnosis based on CT findings was only 36%, compared to 82% with ultrasonography [8]. Hence, estimating the disease or an underlying cause facilitates selecting the appropriate imaging modality, can reduce the number of unnecessary CT scans, and may increase the usefulness of CT scans.

Second, this study revealed that inflammatory responses, as indicated by CRP levels and qSOFA scores, and immunocompromised conditions had limited effect on the CT scan assessment. The CRP values of the CT-finding group were significantly higher than those of the no-CT-finding group. The difference was a median of 0.5 mg/dL, thus, having little impact on clinical practice. In the multivariate analysis, CRP did not significantly affect the assessment of the CT findings, and similarly, other patient aspects such as qSOFA score and immunocompromised status also did not significantly affect it.

One possible reason for the result that in this study only physician-related aspects were associated with CT findings is the restriction of the study population to inpatients without surgical intervention. During hospitalization, physicians might recognize fever earlier and consequently request a CT scan before inflammatory markers such as qSOFA score and CRP level are increased. Although contrast-enhanced CT provides more information in certain cases such as an abscess or an ischemic lesion, the effect of contrast agent use may have been limited, because radiologists assessed all CT images in this study. It is also possible that the value of the physicians’ initial diagnosis was emphasized in this study, because the patient background was well known, including whether the patient was immunosuppressed or not. Furthermore, the subgroup analysis in immunocompromised patients showed that the CRP value was significantly higher in the CT-finding group. This difference might have been caused by delayed symptom recognition, because these patients tend to have fewer physical findings, including fever, compared to patients with normal immunity. However, the number of cases is small in our study, and further studies are needed to confirm these findings.

CT is also a relatively early assessment method in several structured approaches to investigate fever of unknown origin [9–11], albeit with different target diseases. However, as Wright and Auwaerter [9] warned, blind application of such algorithms may result in the performance of an excessive number of examinations. Cunha et al. [12] also indicated that imaging modalities should be selected based on fever of unknown origin and the likely pattern of organ involvement. The opinions articulated by these authors are consistent with our results, demonstrating that the physician’s decisions before the CT examination are valuable.

Another study also emphasized the importance of physician-related aspects for the appropriate utilization of imaging modalities [13]. This study was a questionnaire survey to investigate the appropriateness of diagnostic imaging requests, including CT, magnetic resonance imaging, and X-rays, focusing on full-time radiologists at 165 hospitals with an average of approximately 700 beds in Japan. According to the study, the most frequently inappropriate request for an imaging modality was CT (52–61%). Many of the radiologists also indicated that the imaging examinations were inappropriate in the following cases: craniocaudal expansion of the examination range and whole-body imaging with unclear indication. Their study differs from ours in survey methods and topics, but their findings suggest that the aspect of physicians requesting CT examinations is essential for the appropriate utilization of imaging examinations. The results of their study combined with ours suggest that the physician’s decision before the CT examination affects the usefulness and appropriateness of the CT scan.

Our study has some limitations. First, the study was limited to a single center with a small sample size. The eligible patients were also limited to inpatients without surgical interventions. Hence, our result cannot be generalized to other patients with fever, such as after surgery and in emergency settings. Second, we did not know the exact purpose of the CT use due to the retrospective nature of this cohort study. For example, physicians might request a CT scan not only to diagnose or rule out a specific disease but also to perform this scan due to litigation risks. To evaluate the usefulness, we should consider these purposes separately, but we were not able to do so in this study. This may have affected the proportion of detecting a suspicious CT finding regarding the cause of a fever. Third, some of the “suspicious findings” may constitute asymptomatic incidental findings. To sufficiently evaluate this factor, a control group without fever would be required. We should design a prospective study with more detailed physician-related aspects and increased sample size to resolve these limitations.

## Conclusion

In conclusion, this study suggests that, in the case of hospitalized patients with fever excluding post-surgical procedures, the attending physician’s estimated diagnosis before the CT scan was associated with the assessment of the CT findings. Estimating the cause of fever before the CT examination, regardless of whether the estimation is consistent with the final diagnosis or not, is expected to increase the usefulness of the CT and lead to more appropriate CT utilization, that is, reducing radiation exposure and the number of unnecessary examinations.

## Supplementary Information

Below is the link to the electronic supplementary material.

Supplementary file1 (DOCX 25 KB)
