# Supplementary material for: Usefulness of computed tomography for hospitalized adult patients with fever to investigate cause of fever: single-center, retrospective cohort study
Source: Jpn J Radiol. 2021 May 1;39(8):802–10. doi: 10.1007/s11604-021-01117-5 (PMC8088207; doi:10.1007/s11604-021-01117-5)
Supplement: Supplementary file 1 — Supplementary file1 (DOCX 25 KB) [file 11604_2021_1117_MOESM1_ESM.docx]

**Additional files**

**eTable 1** Characteristics of patients with CT findings regarding the cause of fever

| **Characteristics** | **CT finding** | **Infection-related CT finding** |
| --- | --- | --- |
| N | 114 | 99 |
| Age (years) | 71 (IQR, 55.75-77.0) | 71 (IQR 56-77) |
| Male, sex | 82 (71.9%) | 69 (69.7%) |
| CRP (mg/dL) | 6.75 (IQR, 3.25-12.75) | 6.2 (IQR 3.1-12.6) |
| WBC count (× 10^3^/µL) | 7.95 (IQR, 4.45-12.3) | 7.9 (IQR 4.3-12.3) |
| Creatinine (mg/dL) | 0.87 (IQR, 0.61-1.39) | 0.87 (IQR 0.59-1.38) |
| qSOFA score ≧ 2 | 30 (27.3%) | 26 (27.4%) |
| Ward, ICU | 2 (1.8%) | 2 (2.0%) |
| Immunocompromised conditions^a^ | 60 (52.6%) | 50 (50.5%) |
| HIV | 4 (3.5%) | 3 (3.0%) |
| Anticancer drug | 36 (31.6%) | 29 (29.3%) |
| Steroid use | 20 (17.7%) | 19 (19.2%) |
| Immunosuppressive diseases^b^ | 30 (26.3%) | 29 (29.3%) |
| Diabetes mellitus | 22 (19.3%) | 19 (19.2%) |
| Use of contrast agent in CT | 66 (57.9%) | 57 (57.6%) |
| Presence of an attending physicians’ estimation regarding an infection before the CT scan^c^ | 63 (57.3%) | 53 (55.8%) |
| Departments |  |  |
| Hematology | 21 (18.4%) | 20 (20.2%) |
| Lower Digestive Surgery | 15 (13.2%) | 11 (11.1%) |
| Infectious Disease and Respiratory Medicine | 13 (11.4%) | 12 (12.1%) |
| Nephrology | 10 (8.8%) | 9 (9.1%) |
| Gastroenterology | 10 (8.8%) | 7 (7.1%) |
| Urology | 9 (7.9%) | 8 (8.1%) |
| Upper Digestive Surgery | 8 (7%) | 6 (6.1%) |
| Hepato-Biliary-Pancreatic Surgery | 6 (5.3%) | 5 (5.1%) |
| Cardiology | 5 (4.4%) | 5 (5.1%) |
| Dermatology | 2 (1.8%) | 2 (2.0%) |
| Rheumatology | 2 (1.8%) | 2 (2.0%) |
| Obstetrics and Gynecology | 2 (1.8%) | 1 (1.0%) |
| Endocrinology | 2 (1.8%) | 2 (2.0%) |
| Neurology | 1 (0.9%) | 1 (1.0%) |
| General Medicine | 1 (0.9%) | 1 (1.0%) |
| Neurosurgery | 1 (0.9%) | 1 (1.0%) |
| Cardiovascular Surgery | 1 (0.9%) | 1 (1.0%) |
| Plastic Surgery | 1 (0.9%) | 1 (1.0%) |
| Emergency Department | 1 (0.9%) | 1 (1.0%) |
| Oral and Maxillofacial Surgery | 1 (0.9%) | 1 (1.0%) |
| Orthopedic Surgery | 1 (0.9%) | 1 (1.0%) |
| Otolaryngology | 0 (0%) | 1 (1.0%) |
| Psychiatry | 0 (0%) | 0 (0.0%) |

*CRP* C-reactive protein; *CT* computed tomography; *HIV* human immunodeficiency virus; *ICU* intensive care unit; *IQR* interquartile range; *qSOFA* quick sequential organ failure assessment; *WBC* white blood cell.

^a^ Defined as follows: patients who received any anticancer drug, oral or intravenous steroid therapy, or any immunosuppressive drugs; patients who had immunosuppressive diseases, such as HIV infection or hematologic malignancies.

^b^ This mainly consists of hematologic malignancies.

^c^ We based this on medical records or CT requests.
